# Effects of Filler Distribution on Magnetorheological Silicon-Based Composites

**DOI:** 10.3390/ma12183017

**Published:** 2019-09-18

**Authors:** Sneha Samal, Marcela Škodová, Ignazio Blanco

**Affiliations:** 1 Institute of Physics of Czech Academy of Science, Na Slovance 1999/2, 18221 Prague 8, Czech Republic; 2 The Institute for Nanomaterials, Advanced Technology and Innovation, Technical University of Liberec, Studentska 2, 46117 Liberec, Czech Republic; marcela.skodova@tul.cz; 3 Department of Civil Engineering and Architecture, University of Catania an UdR-Catania Consorzio INSTM, Viale Andrea Doria 6, 95125 Catania, Italy

**Keywords:** MRE composite, iron particles, elastomer, isotropic distribution

## Abstract

The smart materials subclass of magnetorheological elastomer (MRE) composites is presented in this work, which aimed to investigate the influence of filler distribution on surface morphology. Iron particles with sizes ranging from 20 to 150 µm were incorporated into the elastomer matrix and a 30% volume fraction (V%) was chosen as the optimal quantity for the filler amount in the elastomer composite. The surface morphology of MRE composites was examined by 3D micro-computed tomography (µCT) and scanning electron microscopy (SEM) techniques. Isotropic and anisotropic distributions of the iron particles were estimated in the magnetorheological elastomer composites. The filler particle distribution at various heights of the MRE composites was examined. The isotropic distribution of filler particles was observed without any influence from the magnetic field during sample preparation. The anisotropic arrangement of iron fillers within the MRE composites was observed in the presence of a magnetic field during fabrication. It was shown that the linear arrangement of the iron particle chain induced magnetization within the composite. Simulation analysis was also performed to predict the particle distribution of magnetization in the MREs and make a comparison with the experimental observations.

## 1. Introduction

Materials scientists, taking advantage of the best properties of each component and trying to decrease or eliminate their drawbacks, define composites as the material platform of the twenty-first century [1], where two or more components are combined in a single material to give new and previously unattainable combinations of useful properties [2,3].

Magnetorheological elastomer (MRE) composites belong to the category of smart materials, whose properties can be significantly altered by controlled external stimuli, such as stress, temperature, pH, moisture, and electric or magnetic fields [4,5,6,7,8,9]. Elastomers comprising a matrix interspersed with micron-sized ferromagnetic particles are known as MREs, being analogs to magnetorheological (MR) fluids [10]. The rheological properties, namely deformation and flow behavior under stress, of MREs can be altered by the application of an external magnetic field. The characteristic response depends on many factors, including the nature of the matrix, the size, distribution, composition, and quantity of the ferromagnetic particles, and whether the ferromagnetic particles are aligned in chains or randomly dispersed [11]. Besides magnetically controlled smart materials, magnetic composites such as MREs and MR fluids have also demonstrated a high potential for damper applications, especially if the magnetic field generation can be configured to produce lightweight and flexible devices for shock absorption in damping capacitors [12,13,14]. Owing to benefits that include low mass, flexibility, no geometric constraints, cost-effectiveness, and miniaturization, soft actuators configured in a thin film are potentially available for use in various sectors [15,16].

MRE composites consist of two phases, namely magneto-active filler and non-magnetic soft elastomer matrix. The shape and size of the used particles are generally chosen based on the induced magnetization in the MRE composites [17]. The particle–particle interaction within the matrix, when exposed to an external magnetic field, plays a key role in the elastic properties of the composite, thus determining the overall properties and performance of the MRE composites as smart materials [18,19]. The induced magnetic particle–particle interactions strongly depend on the particle distribution within the matrix, thus being influenced by their nearest position and arrangement inside the composites [20,21]. The mechanical properties of the MRE composites, in particular, are highly influenced by the isotropic and/or anisotropic (chain-like, plane-like) distribution of the particles within the matrix [22], depending on the method of preparation [23]. Various research studies have been published concerning the MREs’ properties [24,25,26,27,28], but only a limited number of them speculate about the internal structure of the composites, such as the influence of the particle shape and size and their distribution with and without the presence of an external magnetic field during the fabrication process [29,30,31]. Particle interactions at the microscopic level can alter the macroscopic properties of MRE composites, especially in the presence of an external magnetic field, thus influencing their mechanical properties. The aim of this work was to investigate the 3D internal structure of the MRE composites, with the use or not of a magnetic field during the preparation, by using 3D micro-computed tomography (µCT). Furthermore, the surface morphology and filler distribution within the MRE composites were investigated by scanning electron microscopy (SEM), and both isotropic and anisotropic filler arrangements were evaluated. Finally, to predict the particle distribution in the composite, a simulation mechanism of the magnetic behavior was proposed and compared with the experimental observations.

## 2. Experimental Section

### 2.1. Materials

The first elastomer matrix (A) was obtained by the reaction of a commercial silicone rubber Lukopren N 1522 (Lucební závody, Kolín, Czech Republic) and tetraethyl silicate (TEOS) (Sigma-Aldrich Co., St. Gallen, Switzerland) in the presence of dibutyltin dilaurate (DBTD) (Sigma-Aldrich Co., St. Gallen, Switzerland). The second matrix (B) was prepared by reacting two different silicone rubbers, namely RTV 5532A (Elantas, Collecchio, Italy) and ZA 22 (Mouldlife, Suffolk, UK). Magneto-active fillers such as iron particles (Havel Composites CZ s.r.o., Olomoucky Kraj, Czech Republic) were chosen with an irregular size from 20 to 150 µm (purity >95%).

### 2.2. Techniques

The iron particle distribution and adhesion within the elastomer matrix were examined inside the microstructure of the MRE composites by scanning electron microscopy (Model TM-3000, Hitachi High Technologies Corporation, Tokyo, Japan). The internal structure and 3D images of the MRE composites were observed by micro-computed tomography (Bruker, Kontich, Belgium). The anisotropic distribution of the filler particles within the matrix was estimated through the simulation (MSC MARC software 2018, MSC Software GmbH, Munich, Germany) of the MRE composite in the presence of a magnetic field. The results obtained from the simulation were compared with those obtained experimentally for the MRE samples fabricated in the presence of an external magnetic field. The internal structure of the composites and their mechanism towards a self-assembled structure in isotropic behavior and small chain structures in anisotropic behavior were well examined.

### 2.3. Composite Preparation

**Figure 1:**
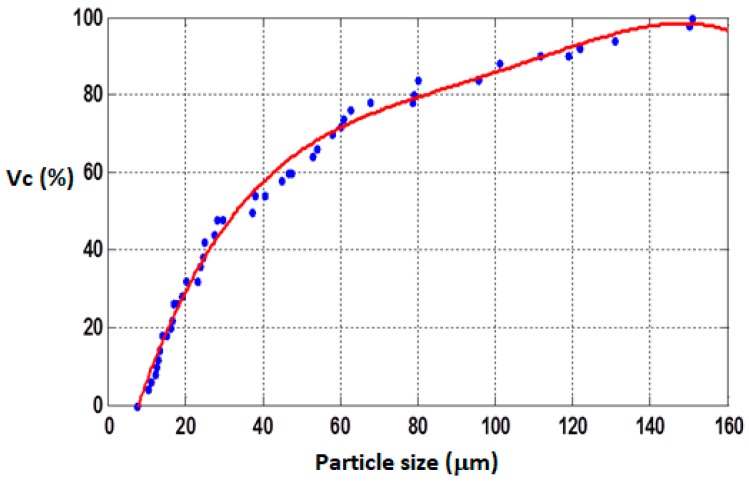
Iron particle distribution within the matrices.

**Figure 2:**
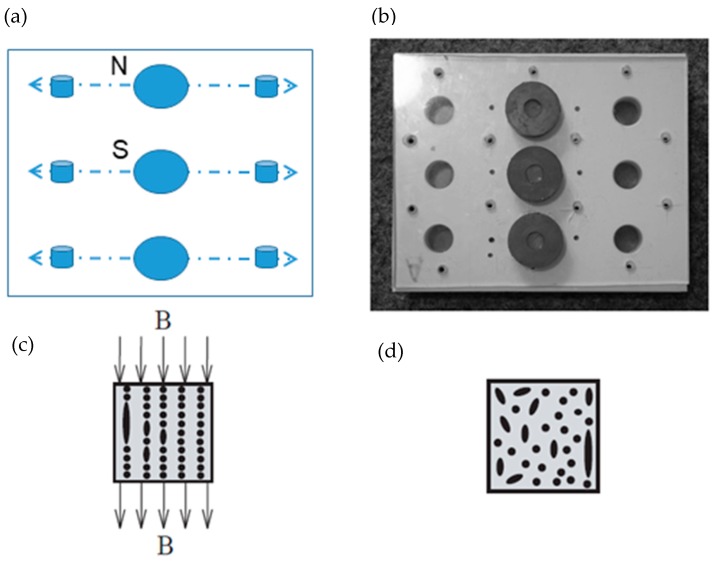
Schematic representation (**a**–**c**) of the MRE composite fabrication with anisotropic filler distribution with the presence of magnetic field and (**d**) isotropic filler distribution without the presence of magnetic field.

According to a suggestion in the literature [32], a volume fraction (V%) equal to 30% was chosen as the optimal filler quantity for the incorporation into the matrix. The elastomers were mixed with the iron powder at room temperature and then held under vacuum (20 KPa) for 12 h. Silicone oil was used as a binder and coupling agent. The obtained composites were removed from the mold after 24 h and then cured at 80 °C for another 24 h. Their composition and polarizable character are reported in Table 1. The iron particle distribution is presented in Figure 1, showing an average particle size of approximately 50 µm. The isotropic and anisotropic distributions of the fillers within the MRE during fabrication are presented in the schematic diagram (Figure 2a–d).

## 3. Results and Discussion

### 3.1. Internal Image of the MRE Composite using 3D Image Acquisition

**Figure 3:**
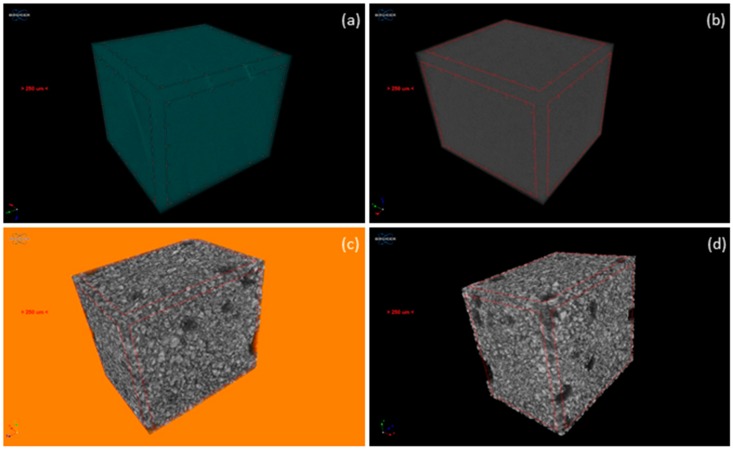
3D internal image of the elastomer matrices (**a**,**b**) and the MRE composites (**c**,**d**) with isotropic filler distribution.

The internal image of the elastomer matrices (A and B) and their respective MRE composites with isotropic filler distribution are reported in Figure 3, where their 3D constructions with a representative volume of the elements are shown.

**Figure 4:**
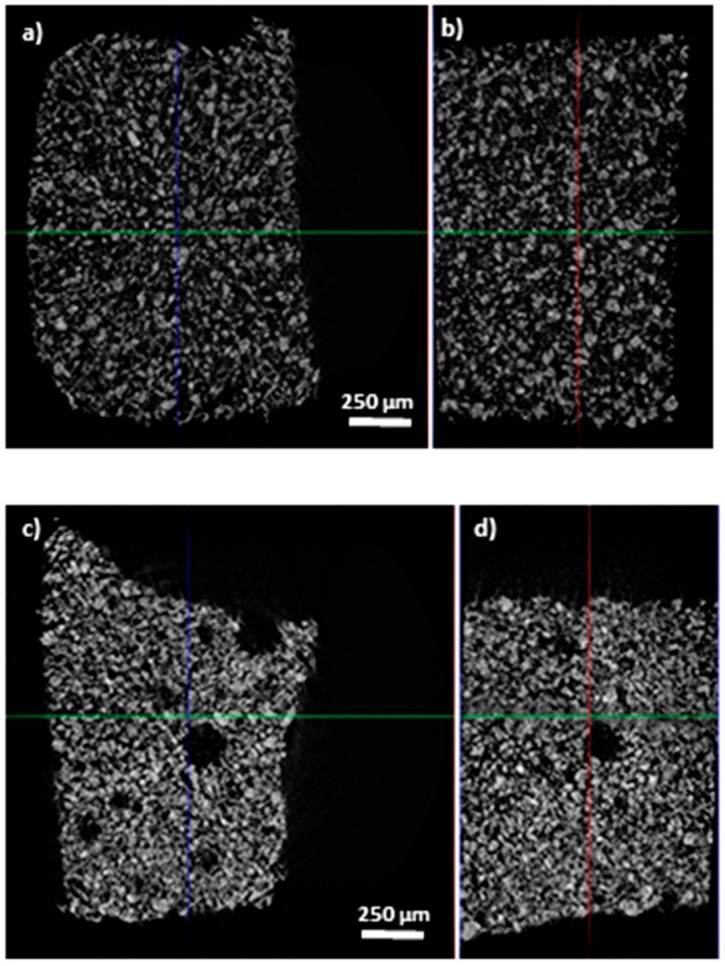
2D cut view of the MRE composite with (**a**,**b**) isotropic filler distribution shows homogenous structure without any porosity (elastomers A and B); (**c**,**d**) anisotropic distribution shows more voids due to magnetic field (elastomers A and B).

The internal areas show the uniform embodiment of iron particles within the composite’s matrix; however, porosities can be also observed in some of them. Slices from the overall 3D images are presented in Figure 4, where the 2D image of the XY pattern shows the isotropic and anisotropic effects within the MRE composites. The isotropic one resulted in a homogenous and uniform distribution of the filler particles with the frame of the MRE composites (Figure 4a,b), whereas for the anisotropic behavior the influence of the magnetic field results in voids inside the composites (Figure 4c,d).

As shown, the particle distribution is in the micron scale and the composite density obeys the following equation [32]:

(1) $$\rho_{c}V_{c}=\rho_{m}V_{m}+\rho_{f}V_{f}+\rho_{v}V_{v},$$

where *ρ_c_* and *V_c_* represent the composite density and volume; *ρ_f_* and *V_f_* are the filler density and volume; *ρ_m_* and *V_m_* are the matrix density and volume; and *ρ_v_* and *V_v_* the void density and volume, respectively. When the void density is negligible, *ρ_v_* ~ 0, and thus Equation (1) can be modified into the following equation:

(2) $$\rho_{c}V_{c}=\rho_{m}V_{m}+\rho_{f}V_{f}.$$

Because the volume of the filler amount remains constant, the volume of the matrix varies based on the filler content, as follows:

(3) $$\rho_{c}V_{c}=(\frac{1}{\rho_{f}}-\rho_{f})V_{m}+\rho_{f}V_{f},$$

and, depending upon the void volume, Equation (3) can be further modified. Thus, the overall quality of the internal structure is a function of the filler distribution within the MRE composite’s matrix.

### 3.2. Distribution of Iron Particles within the MRE Composite

**Figure 5:**
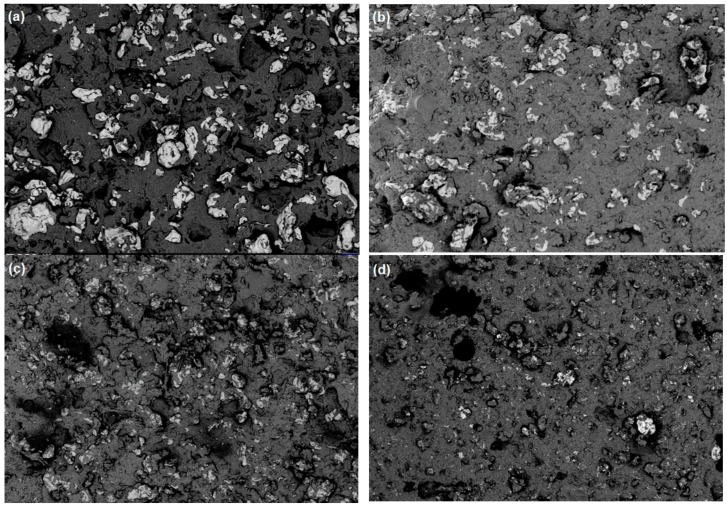
Scanning electron microscopy (SEM) images of the MRE composites A and B without the presence of a magnetic field (**a**,**b**), and prepared in the presence of a magnetic field (**c**,**d**).

In Figure 5, SEM images of the MRE composites, prepared in the presence (Figure 5a,b) and absence (Figure 5c,d) of a magnetic field, are shown. When we applied a magnetic field during the preparation, we observed an isotropic distribution without any porosity, whereas for the composite prepared without this application, anisotropic distribution was evident. In this latter composite, porosity is observed towards its edge, which may be due to the aligned magnetic forces, resulting in the creation of voids.

**Figure 6:**
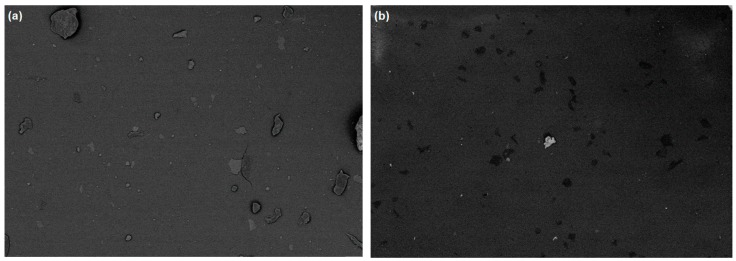
SEM images of the surface (height = 0 mm) of the composites A (**a**) and B (**b**).

In order to better analyze the iron particle distribution within the elastomeric matrices, the samples were cut at various heights from the top, middle, and bottom positions. SEM images of the top layer showed less particle distribution within the composites (Figure 6a,b).

**Figure 7:**
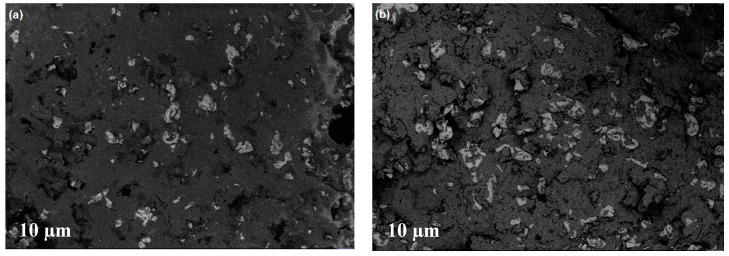
SEM images of the inner surface (height = 0.8 mm) of the composites A (**a**) and B (**b**).

**Figure 8:**
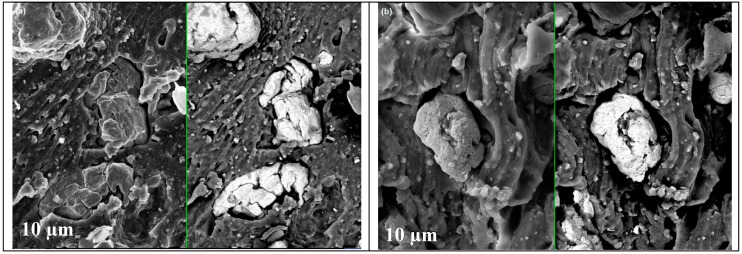
SEM images showing filler–matrix adhesion for the composites A (**a**) and B (**b**).

By contrast, a better and quite uniform distribution was observed in the middle and bottom layers. In particular, small fibril chains of particles (Figure 7a,b) and good filler–matrix adhesion (Figure 8a–b), despite some aggregation phenomena, are shown.

### 3.3. Analysis of the Iron Particle Distribution along the MRE Composites

**Figure 9:**
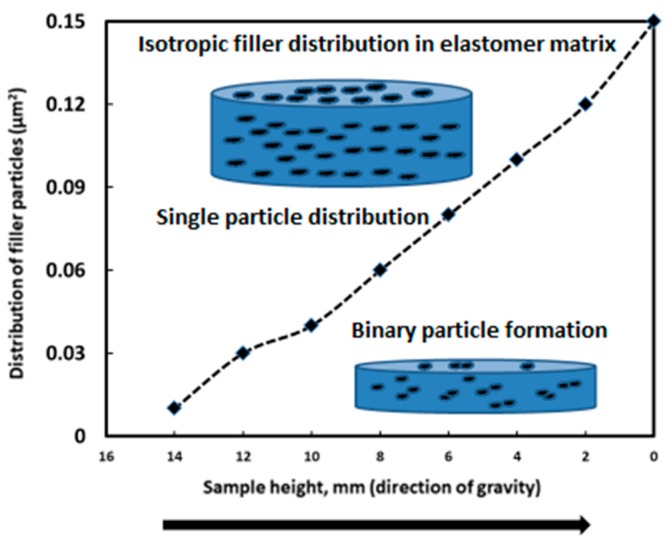
Distribution of filler with single and binary behavior as a function of the height of the MRE composite.

The distribution profile of iron particles as a function of the height of the MRE composite was calculated, showing the filler mostly settled down towards the base of the composite, whereas fewer particles were present towards the upper position. The single particle distribution in the elastomeric composite versus the binary particle distribution in its surface area is plotted in Figure 9.

**Figure 10:**
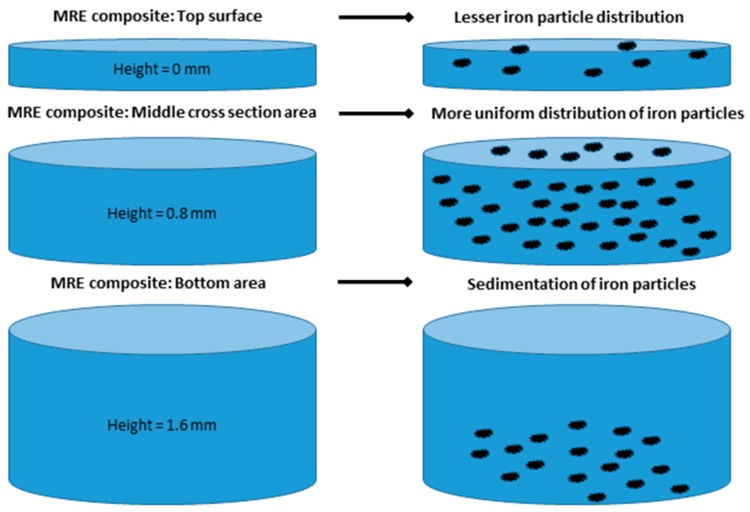
Iron particle distribution within various heights of the MRE composite from the top to the bottom areas.

The binary particle distribution is more pronounced along the base of the composite and the volume fraction is less in comparison to the single isotropic filler distribution. Cutting the MRE composite into three pieces (top, middle, and bottom positions) led us to hypothesize a schematic filler distribution within the different composite layers (Figure 10).

The distribution of iron particles within the MRE composites showed a smaller amount at the surface and a uniform distribution within the middle cross-section area. Finally, the bottom area showed the binary combination of particles settled on the base of the composite.

### 3.4. Simulation Analysis and Anisotropic Filler Distribution within the MRE Composite

**Figure 11:**
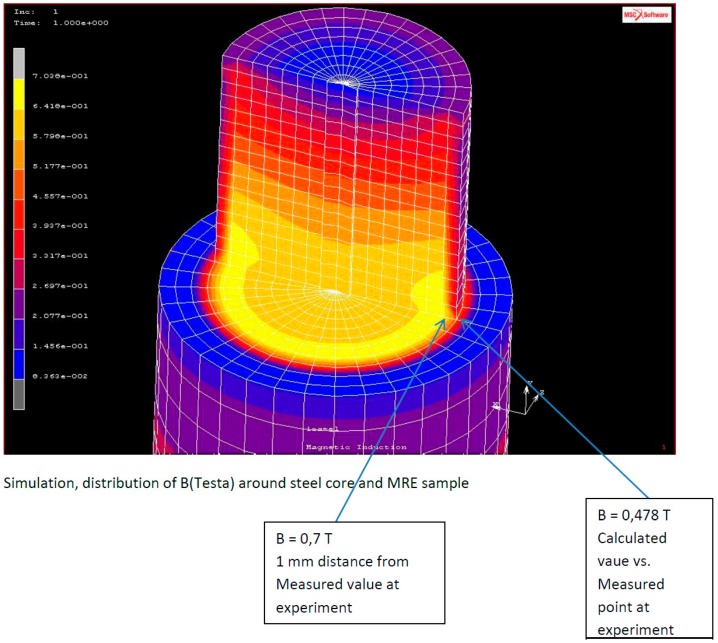
Distribution of magnetic induction in the MRE composite.

A simulation analysis in the presence of a magnetic field was carried out, and the magnetic induction generated in the MRE composite, at outer and inner positions, is presented in Figure 11.

**Figure 12:**
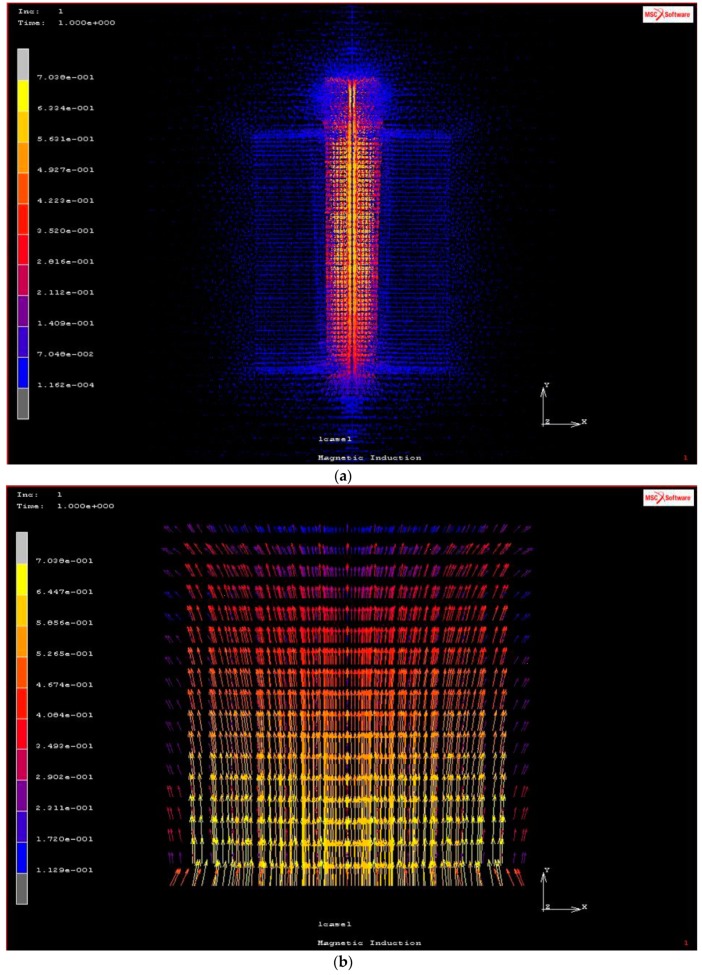
Magnetic induction distribution around the coil and sample on the top (**a**), and inside the MRE composite (**b**).

The magnetic induction B calculated at the outer and inner edges show values of 0.478 and 0.700 T, respectively (Table S1, Supplementary Materials). The magnetic induction distribution around a coil, with a sample position on the top, and the magnetic induction inside the MRE composite are reported in Figure 12, respectively, showing a linear uniform flow of the magnetic field from the lower to the upper regions of the composite. Furthermore, the theoretical prediction of the magnetic force and induction at various magnetic current values and various positions of compression values are reported in Figures S1 and S2 (Supplementary Materials).

The presence of a magnetic field leads to an anisotropic distribution of the iron particles within the MRE composite, resulting in small linear chains of particles such as fibrils arranged perpendicularly within the normal axis matrix [33]. In addition, the presence of the magnetic field induces magnetic forces to create small voids along the edge of the composite. Upon anisotropic distribution, an alignment of iron particles within the matrix was observed and the dipolar axis related to the magnetization followed one direction, which is different from the isotropic distribution in which the magnetic dipolar forces were randomly oriented within the composite. The internal forces between two particles result in the combined coherent forces due to the surface energy effect. Otherwise, self-assembled structures lead towards the magnetization in the MRE composite.

**Figure 13:**
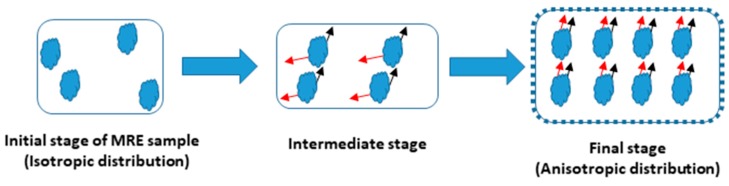
Iron particle distribution before and after the application of the magnetic field.

A schematic diagram of this phenomenon is shown in Figure 13:

1. The initial stage of the MRE composite, without the presence of a magnetic field, shows a random distribution of iron particles within the matrix;

2. Applying a magnetic field, perpendicular to the surface of the composite, results in the development of dipolar magnetization in the particles;

3. Increasing the magnetization, a strong alignment within the fillers results in an anisotropic distribution of the particles [34,35,36,37,38,39].

## 4. Conclusions

The internal structure of MRE composites was studied, showing isotropic and anisotropic distributions of the iron particles within the elastomeric matrix, whose quantity and nature (magneto-active) influenced the mechanical and magnetic properties of the obtained material. The effect of particle distribution within the composites was investigated at various heights, showing a different behavior from the top to the bottom of the samples. The top surface showed a lesser distribution of the particles; however, the middle and bottom surfaces showed particle sedimentation within the composite volume for micron-sized ones. The sedimentation was less for smaller-sized particles, resulting in a more uniform distribution. The anisotropic filler alignment was explained on the basis of the iron particle interaction, due to the magnetic induction. This latter, as it has been seen, leads to the formation of particle chains as fibrils within the matrix. Thus, the dipolar interaction between two particles within an isotropic distribution presentation plays a key role in the magnetic behavior of the MRE composite. The maximum magnetic induction of 0.659 T was achieved at a magnetic current value of 7 A for the MRE composite during the compression analysis.

## Figures and Tables

**Table 1:** Composition and polarizable character of the magnetorheological elastomer (MRE) composites.

Sample	Filler	Matrix	Polarized
A	0	N1522	no
A1	30 V%	N1522	no
A2	30 V%	N1522	yes
B	0	ZA22	no
B1	30 V%	ZA22	no
B2	30 V%	ZA22	yes

## Supplementary Materials

The following are available online at https://www.mdpi.com/1996-1944/12/18/3017/s1. Figure S1: Magnetic forces theoretical prediction using various magnetic currents as a function of compression shift position, Figure S2: Magnetic inductions theoretical prediction using various magnetic currents as a function of compression shift position, Figure S3: Carbonyl iron (CI) particles of size range 1–5 μm within the MRE composite, Table S1: Measured values of magnetic force and induction at various current and compression position of the magnetorheological elastomer (MRE) composites.
