# Supplementary material for: Effects of Filler Distribution on Magnetorheological Silicon-Based Composites
Source: Materials (Basel). 2019 Sep 18;12(18):3017. doi: 10.3390/ma12183017 (PMC6766335; doi:10.3390/ma12183017)
Supplement: Supplementary file 1 [file materials-12-03017-s001.pdf]

materials

Article

Effects of Filler Distribution on Magnetorheological Silicon-Based Composites

Sneha Samal [1,*], Marcela Škodová [2], and Ignazio Blanco [3,*]

[1] Institute of Physics of Czech Academy of Science, Na Slovance 1999/2, 18221 Prague 8, Czech Republic
[2] The Institute for Nanomaterials, Advanced Technology and Innovation, Technical University of Liberec, Studentska 2, 46117 Liberec, Czech Republic; marcela.skodova@tul.cz
[3] Department of Civil Engineering and Architecture, University of Catania an UdR-Catania Consorzio INSTM, Viale Andrea Doria 6, 95125 Catania, Italy
* Correspondence: samal@fzu.cz (S.S.); iblanco@unict.it (I.B.)

Received: 17 July 2019; Accepted: 12 September 2019; Published: 18 September 2019

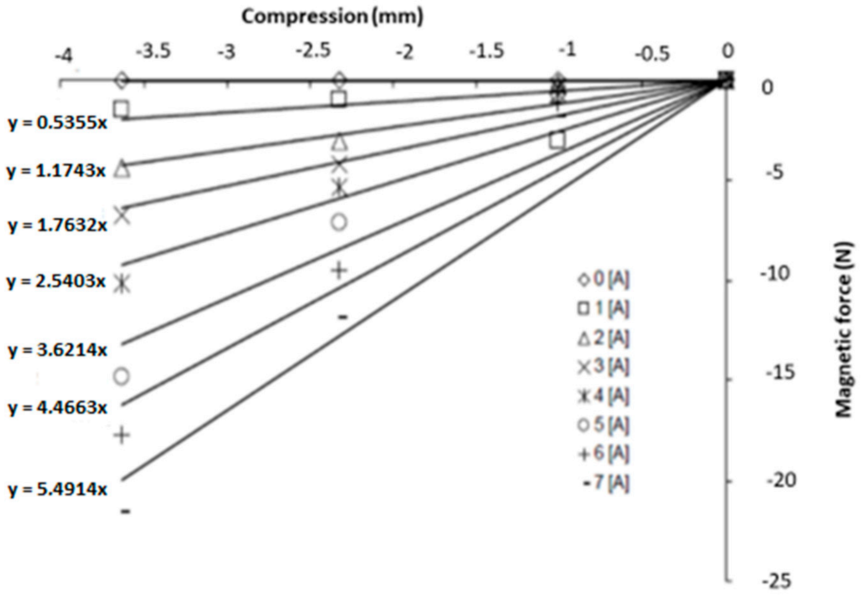

Figure S1. Magnetic forces theoretical prediction using various magnetic currents as a function of compression shift position.

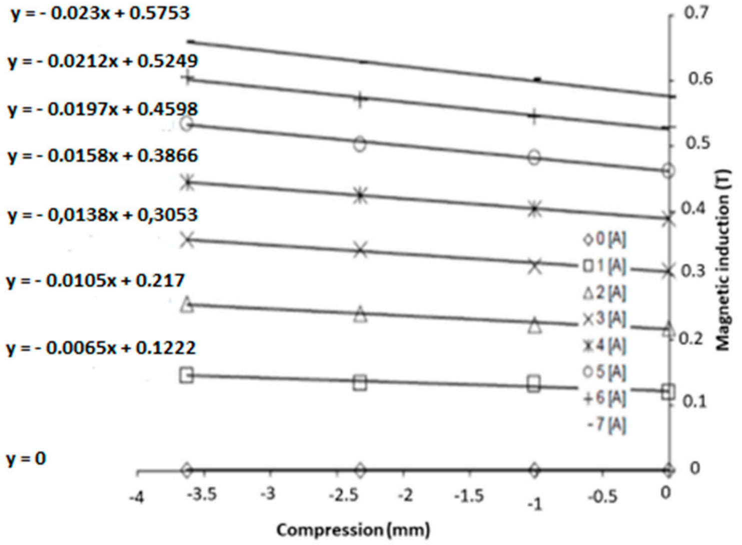

Figure S2. Magnetic inductions theoretical prediction using various magnetic currents as a function of compression shift position.

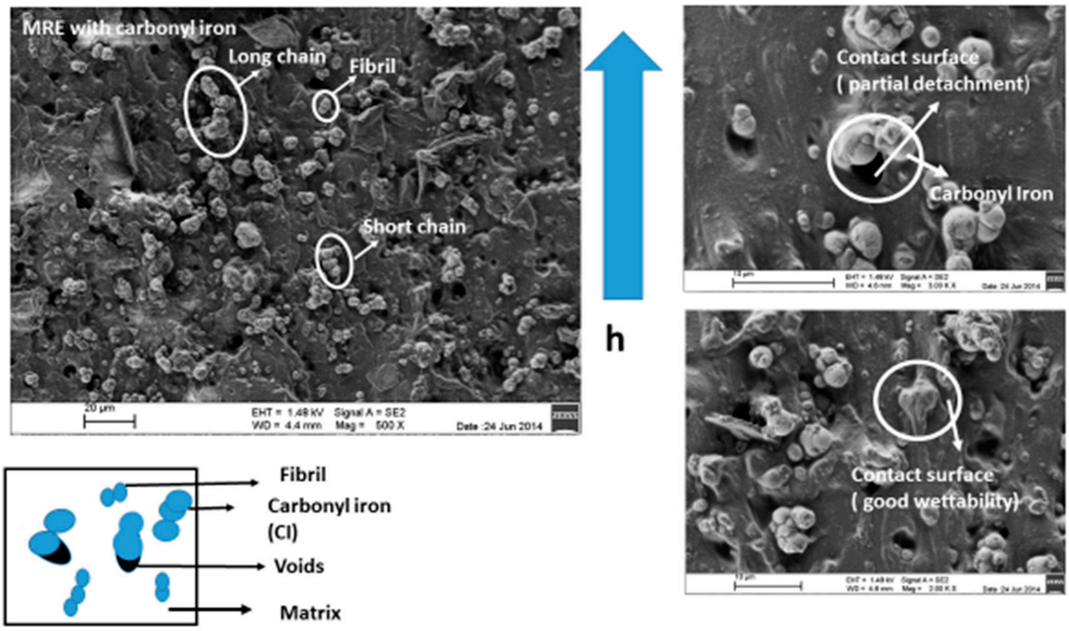

Figure S3. Carbonyl iron (CI) particles of size range 1–5 μm within the MRE composite.

Table S1. Measured values of magnetic force and induction at various current and compression position of the magnetorheological elastomer (MRE) composites.

Current / A	Shift position / mm	Magnetic Force / N	Magnetic Induction / T
0	0	0	0
0	−1.01	0	0
0	−2.32	0	0
0	−3.63	0	0
1	0	0	0.120
1	−1.01	−3	0.133
1	−2.32	−0.96	0.135

1	−3.63	−1.44	0.146
2	0	0	0.219
2	−1.01	−0.16	0.225
2	−2.32	−3.05	0.241
2	−3.63	−4.34	0.256
3	0	0	0.308
3	−1.01	−0.32	0.314
3	−2.32	−4.18	0.340
3	−3.63	−6.75	0.355
4	0	0	0.387
4	−1.01	−0.64	0.402
4	−2.32	−5.31	0.423
4	−3.63	−10.13	0.444
5	0	0	0.460
5	−1.01	−0.64	0.478
5	−2.32	−7.08	0.502
5	−3.63	−14.83	0.533
6	0	0	0.528
6	−1.01	−1.12	0.544
6	−2.32	−9.48	0.570
6	−3.63	−17.12	0.605
7	0	0	0.574
7	−1.01	−1.84	0.601
7	−2.32	−11.84	0.627
7	−3.63	−21.54	0.700

© 2019 by the authors. Submitted for possible open access publication under the terms and conditions of the Creative Commons Attribution (CC BY) license (http://creativecommons.org/licenses/by/4.0/).
